# Enhanced Synaptic Behaviors in Chitosan Electrolyte-Based Electric-Double-Layer Transistors with Poly-Si Nanowire Channel Structures

**DOI:** 10.3390/biomimetics8050432

**Published:** 2023-09-18

**Authors:** Dong-Hee Lee, Hwi-Su Kim, Ki-Woong Park, Hamin Park, Won-Ju Cho

**Affiliations:** 1 Department of Electronic Materials Engineering, Kwangwoon University, Gwangun-ro 20, Nowon-gu, Seoul 01897, Republic of Korea; zpsxlzje@naver.com (D.-H.L.); hwisu0811@naver.com (H.-S.K.); wwoong97@naver.com (K.-W.P.); 2 Department of Electronic Engineering, Kwangwoon University, Gwangun-ro 20, Nowon-gu, Seoul 01897, Republic of Korea; parkhamin@kw.ac.kr

**Keywords:** nanowire channel, chitosan electrolyte, electric double layers, synaptic transistor, neuromorphic computing

## Abstract

In this study, we enhance the synaptic behavior of artificial synaptic transistors by utilizing nanowire (NW)-type polysilicon channel structures. The high surface-to-volume ratio of the NW channels enables efficient modulation of the channel conductance, which is interpreted as the synaptic weight. As a result, NW-type synaptic transistors exhibit a larger hysteresis window compared to film-type synaptic transistors, even within the same gate voltage sweeping range. Moreover, NW-type synaptic transistors demonstrate superior short-term facilitation and long-term memory transition compared with film-type ones, as evidenced by the measured paired-pulse facilitation and excitatory post-synaptic current characteristics at varying frequencies and pulse numbers. Additionally, we observed gradual potentiation/depression characteristics, making these artificial synapses applicable to artificial neural networks. Furthermore, the NW-type synaptic transistors exhibit improved Modified National Institute of Standards and Technology pattern recognition rate of 91.2%. In conclusion, NW structure channels are expected to be a promising technology for next-generation artificial intelligence (AI) semiconductors, and the integration of NW structure channels has significant potential to advance AI semiconductor technology.

## 1. Introduction

Following the rapid development of artificial intelligence (AI) technology, there is an increasing demand for innovative solutions to process exponentially growing amounts of unstructured data [1,2]. Conventional computations based on the von Neumann architecture face significant challenges in dealing with massive and complex information owing to the high power consumption and bottlenecks caused by the physical separation of processing and storage units [3,4,5]. To overcome these problems, neuromorphic architecture, which is a more efficient low-power computing system inspired by the human brain, has been devised [6]. Unlike conventional computing, the human brain possesses unique abilities for simultaneous computation and storage of complex information at ultralow power levels (~20 W) [7]. This exceptional efficiency is achieved by the brain’s massively parallel, reconfigurable, and fault-tolerant nervous system, comprising billions of neurons and trillions of synapses [8,9,10]. Among these components, synapses play a crucial role in modulating learning and memory functions by adjusting the connection strengths between neurons [11]. Consequently, the development of artificial synaptic devices becomes a vital step in realizing efficient neuromorphic computing systems. To implement artificial synapses, synaptic transistors based on various ion-conductive electrolytes have been actively explored [12,13,14,15]. These devices can emulate synaptic functions by mimicking the behavior of neurotransmitters through mobile ions in the electrolyte. Moreover, ion-conducting electrolytes offer large capacitance (~1 μF/cm^2^) owing to the formation of an electric double layer (EDL) on the nanogap scale. As a result, these synaptic transistors can modulate synaptic plasticity by controlling the channel conductivity through mobile ions, even with low driving voltages [16,17]. Chitosan, extracted from chitin, the second-most prevalent biopolymer on Earth, is one such EDL electrolyte with advantages of high biodegradability and renewability. Its high-density mobile protons enable a high gate capacitance (>1.0 μF/cm^2^) and effective emulation of synaptic behavior [13,18,19,20]. However, despite these benefits, chitosan has chemical and mechanical weaknesses as an organic polymer material, which previously limited the fabrication of synaptic transistors with various structures using lithography processes. To address these limitations, we proposed a solution by stacking a biocompatible high-*k* Ta_2_O_5_ film as a barrier layer with a chitosan electrolyte, enabling lithography processes and facilitating the implementation of various chitosan-based synaptic transistors [21]. Additionally, we harnessed the advantages of nanowire (NW) channels in transistors, providing exceptional charge control, increased gate capacitance, and low off-state current due to the high surface-area-to-volume ratio of the nanowire geometry [22,23,24].

In this study, we fabricated a chitosan electrolyte-based synaptic transistor with a polycrystalline silicon (poly-Si) nanowire (NW) channel, leveraging the synergistic effect of the EDL and NW channels to enhance synaptic properties. The NW structure was efficiently formed by electrospinning, an inexpensive and simple process without vacuum equipment. Specifically, we prepared poly-Si NWs by transferring polyvinylpyrrolidone (PVP) nanofiber (NF) template patterns to the poly-Si layer through dry etching. The proposed synaptic transistor with poly-Si NW successfully implements and improves essential synaptic behaviors, including excitatory post-synaptic current (EPSC) facilitated by single, paired, and multi-spike events, as well as potentiation/depression characteristics. Furthermore, we conducted simulations of an artificial neural network (ANN) for recognition tests using the handwritten Modified National Institute of Standards and Technology (MNIST) dataset. Comparing the results with poly-Si film-type channel synaptic transistors based on the chitosan electrolyte, we confirm that the poly-Si NW-type synaptic transistors exhibit even more improved synaptic properties and recognition rates.

## 2. Materials and Methods

### 2.1. Formation of Poly-Si NW Channel

We fabricated the NW channel structure by transferring the pattern of the PVP NFs template onto the poly-Si thin film. To form the poly-Si NW, an undoped poly-Si film with a thickness of 100 nm was deposited using low-pressure chemical vapor deposition (LPCVD) at 530 °C on the SiO_2_/p-Si substrate. The PVP precursor solution was prepared by stirring the PVP powder (100 mg; MW ≈ 1,300,000, Sigma-Aldrich, Saint Louis, MO, USA) dissolved in ethanol (1.5 mL; ≥99.7%) at 800 rpm for 2 h at room temperature. Subsequently, the prepared PVP precursor solution was electrospun onto the poly-Si thin film to form the PVP NFs template. A schematic diagram of the electrospinning equipment is shown in Figure S1 (Supporting Information). This equipment consists of a syringe pump that supplies the solution at a constant flow rate, a grounded copper collector, a metal spinning needle that forms a Taylor cone by applying high voltage, and a high-voltage power supply. The syringe was horizontally clamped to a syringe pump (NE-1000; New Era Pump Systems, Farmingdale, NY, USA) set at a flow rate of 0.4 mL/h. The metal spinning needle and the grounded collector were positioned 20 cm apart, and a voltage of 20 kV was applied to the needle. The electrospinning process lasted for about 1 min while maintaining a temperature of 25 °C and humidity of 25% around the equipment. After electrospinning, the PVP NFs on the poly-Si thin film were calcined for 30 min at 300 °C in ambient air using a resistance-heated furnace. This calcination process provided thermodynamic stability and sufficient adhesion to the thin films of the PVP NFs by removing the internal solvent. As a result, the PVP NFs could function as etch masks for reactive ion etching (RIE). The finally formed PVP NFs had a random network structure with an average diameter of approximately 450 nm. Subsequently, the exposed poly-Si thin films not covered by PVP NFs were etched using SF_6_ plasma. Finally, residual PVP NFs were completely removed by a sulfuric acid–hydrogen peroxide mixture (SPM) solution, resulting in the formation of the poly-Si NW channel. The process sequence for poly-Si NW fabrication is presented in Figure S2 (Supporting Information).

### 2.2. Preparation of Chitosan Electrolyte Solution

Chitosan electrolyte solution, which plays an important role in mimicking synaptic behaviors, was prepared as follows. First, 2 wt% chitosan powder (deacetylation degree > 75%) was added to deionized water diluted with 2 wt% acetic acid (purity > 99%). The solution was then completely dissolved by magnetic stirring at 800 rpm for 6 h at 50 °C. Finally, the chitosan electrolyte solution was obtained by filtering the solution using a polytetrafluoroethylene syringe filter with a 5 μm pore size (Whatman International Ltd., Maidstone, UK).

### 2.3. Fabrication of Synaptic Transistor

A p-Si substrate, on which a 100 nm-thick SiO_2_ layer was thermally grown, served as the starting material and was cleaned using a Radio Corporation of America (RCA) cleaning process. For the channel layer, an undoped poly-Si film with a thickness of 100 nm was deposited through LPCVD. To form N^+^-type source/drain (S/D) regions, the S/D area was doped using the solid-phase diffusion (SPD) method, utilizing phosphosilicate glass (PSG) coating and rapid thermal annealing (RTA). Subsequently, an active channel region (width/length = 160 μm/120 μm) of poly-Si was defined through a photolithography process, followed by an etching step using a Si etchant. The poly-Si NW channel was then formed by transferring the PVP NFs template using RIE. For the formation of the chitosan electrolyte EDL, the prepared chitosan solution was coated at 6000 rpm for 30 s, dried for 24 h under ambient conditions, and oven-baked at 130 °C for 10 min, resulting in a thickness of 130 nm. As a chemical and mechanical reinforcement barrier layer, an 80 nm-thick high-*k* Ta_2_O_5_ film was deposited on the chitosan EDL using a radio frequency (RF) magnetron sputtering system. For the top gate electrode, a 150 nm-thick layer of Al was deposited using an E-beam evaporator and patterned through a lift-off process. Finally, contact holes for S/D measurements were opened using RIE. Figure S3 illustrates the roughness profile and scanning electron microscopy (SEM) image of the poly-Si NW channel (Supporting Information).

### 2.4. Characterization

The surface morphology of the poly-Si NW was examined using SEM (Sirion 400, FEI Company, Hillsboro, OR, USA). The electrical characteristics and synaptic behaviors of the fabricated synaptic transistor were measured in a dark box to protect against external electrical/optical noise. Various measurements were evaluated using an Agilent 4156B Precision Semiconductor Parameter Analyzer (Hewlett-Packard Co., Palo Alto, CA, USA). The synaptic pulses were applied through the Agilent 8110A Pulse Generator (Hewlett-Packard Co., Palo Alto, CA, USA). The characteristics of the NW-type synaptic transistor were compared with those of the film-type device.

## 3. Results and Discussion

**Figure 1:**
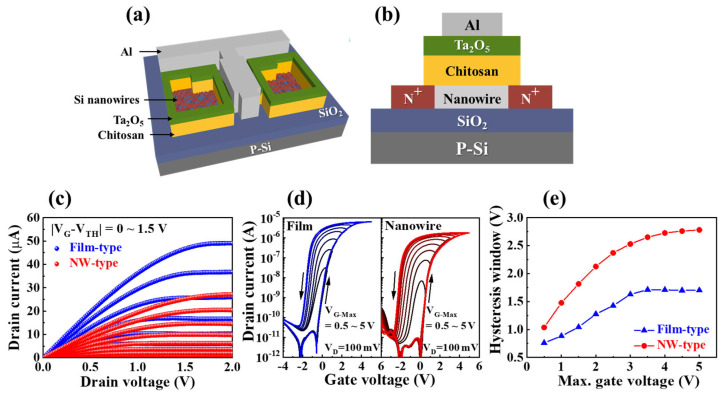
Schematics of (**a**) a three-dimensional structure and (**b**) a two-dimensional vertical cross-section of the polycrystalline silicon nanowire (NW)-type chitosan electric double layer (EDL)-based synaptic transistor. The film-type device has the same structure except for the channel. (**c**) Output characteristic curves (I_D_–V_D_) of the film-type and NW-type chitosan EDL-based synaptic transistors (V_G_-V_TH_ = 0–1.5 V in 0.15 V increments). (**d**) Double-sweep transfer characteristic curves (I_D_–V_G_) obtained by varying the V_G-max_ sweep range (V_G-max_ = 0.5–5 V in 0.5 V increments). (**e**) Extracted hysteresis window for each corresponding double-sweep transfer curve.

Figure 1a,b depicts the schematic of the structure and cross-section view, respectively, of the proposed chitosan EDL poly-Si NW-channel synaptic transistor. In Figure 1c, the output characteristics curves (I_D_–V_D_) of the film- and NW-type synaptic transistors are illustrated. The I_D_ was measured while sweeping V_D_ from 0 V to 2 V within the range of |V_G_-V_TH_| = 0–1.5 V. Both devices exhibite linearly increasing I_D_ at low V_D_ and stable saturated output characteristics at high V_D_, indicating well-formed ohmic contacts at the source and drain [25]. Notably, the NW-type device demonstrated lower I_D_ due to a reduced current path, which can significantly contribute to reducing power consumption in artificial synaptic devices.

For continuous modulation of synaptic weights in an EDL-based synaptic transistor, a continuous variation in the intensity of internal ion polarization is necessary. Figure 1d validates the continuous polarization characteristics of ions through a DC sweep. The film- and NW-type synaptic transistors were gradually increased from 0.5 V to 5 V for maximum V_G_, and the transfer characteristics (I_D_–V_G_) were measured in a double-sweep mode. As shown in Figure 1e, the hysteresis window of each transfer characteristic curve corresponding to the maximum V_G_ is also shown. As the maximum V_G_ increases, the degree of polarization of internal protons in the chitosan layer intensifies, requiring a higher negative voltage to depolarize back to the initial state. The hysteresis window exhibits a continuous increase with maximum V_G_, demonstrating the feasibility of implementing synaptic behavior in the proposed device.

Additionally, the film-type synaptic transistor shows a lower hysteresis window and faster saturation for all maximum V_G_ compared to the NW-type device. This result is attributed to the higher volume-to-area ratio of the NW-type channel compared to the film-type channel. Figure S4 in the Supporting Information shows a schematic diagram for proton migration in the film- and NW-type devices. The NW structure, surrounded by the chitosan electrolyte, is more strongly affected by the protons of the electrolyte, enabling a more effective modulation of the channel conductivity within the same gate voltage range [25,26,27].

**Figure 2:**
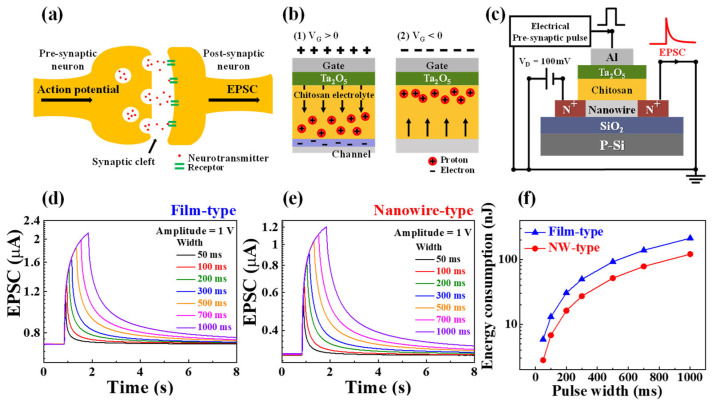
Schematic diagram illustrating (**a**) the structural configuration of biological synapses in the brain, and (**b**) migration of protons in the chitosan electrolyte depending on the V_G_. (**c**) Schematic of the measurement circuit for excitatory post-synaptic current (EPSC). EPSC characteristics for varying pulse widths in (**d**) film-type and (**e**) NW-type chitosan EDL-based synaptic transistors. (**f**) Energy consumption properties corresponding to pulse widths in the film- and NW-type chitosan EDL-based synaptic transistors.

In the human brain, the fundamental function of a biological synapse is to transmit spike-shaped signals (stimuli) from the pre-synapse to the post-synapse, allowing for the modulation of synaptic weights and the determination of neuronal firing [28]. Figure 2a depicts a schematic diagram of a biological synapse within the brain, which plays a crucial role in signal transmission between pre- and post-synaptic neurons. The signal transmission occurs as neurotransmitters (K^+^, Na^+^ ions) are conveyed through the synaptic cleft.

In the chitosan EDL-based synaptic transistor, the internal protons of the chitosan electrolyte act as neurotransmitters, emulating the transmission of spike signals from pre- to post-synapse. Figure 2b shows the migration of the protons in the chitosan electrolyte depending on the V_G_. At positive V_G_, the protons move toward the channel and induce channel carriers, forming a path for current flow. Conversely, at negative V_G_, the protons move to the other side of the channel, reducing the channel carriers. As illustrated in Figure 2c, the fabricated synaptic transistor measures the EPSC to represent the current flowing through the channel in response to positive gate stimulation. When a pre-synaptic pulse is applied to the gate while V_D_ is maintained at 100 mV, the current initially increases and then slowly decreases due to the gradual re-diffusion of protons. The EPSC can be effectively adjusted by modifying the intensity, width, and frequency of the pre-synaptic pulse.

Figure 2d,e presents the measured results of EPSCs concerning the pulse width for the film- and NW-type synaptic transistors. The synaptic pre-pulse amplitude was consistently set at 1 V, and the EPSC value showed a gradual increase as the pulse width increased. Moreover, the EPSC value gradually decreased after the pulse application ended, with a wider pulse width resulting in a longer duration of EPSC.

Furthermore, Figure 2f displays the energy consumption per pulse width for the film- and NW-type devices. The energy consumption was calculated as *I_peak_* × *t* × V_D_, where *I_peak_*, *t*, and V_D_ represent the peak EPSC current, pulse width, and drain voltage, respectively [29,30]. The NW-type device demonstrated lower energy consumption compared with that of the film-type device. Notably, at a pulse width of 50 ms, the energy consumption of the NW-type device was ~2.1 times lower than that of the film-type device (the NW-type: 2.7 nJ and the film-type: 5.8 nJ). These results indicate that the NW-structure channel effectively reduces energy consumption, making it a promising approach for energy-efficient synaptic devices.

Paired-pulse facilitation (PPF) is a critical form of short-term plasticity in biological synapses, crucial for processing temporal information in visual or auditory signals [31,32]. PPF refers to the phenomenon where the second EPSC is transiently facilitated when two pulses are applied consecutively, with the facilitation becoming stronger as the inter-pulse interval decreases. This phenomenon arises due to the accumulation of mobile protons at the electrolyte/channel interface following the first pre-synaptic spike. When a second spike is applied with a sufficiently short inter-spike interval (∆t_inter_), the continuous accumulation of mobile protons at the interface leads to an increase in channel conductivity.

**Figure 3:**
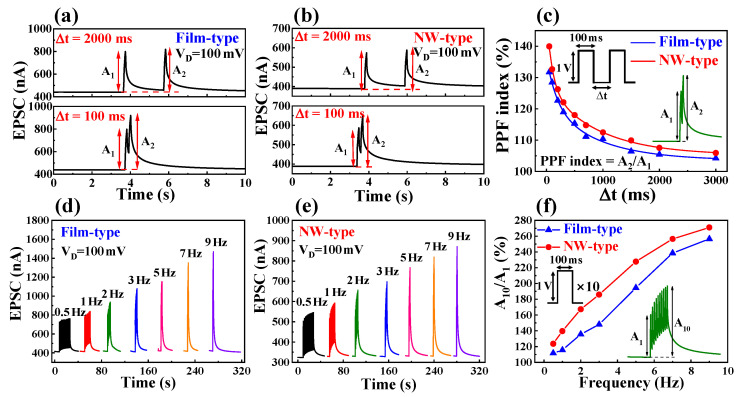
EPSCs facilitated by a paired spikes at 100 ms and 2000 ms intervals of (**a**) film-type and (**b**) NW-type chitosan EDL-based synaptic transistors. (**c**) Paired-pulse facilitation (PPF) index (A_2_/A_1_) of the film-type and NW-type chitosan EDL-based synaptic transistors as a function of Δt_inter_ (100 to 3000 ms) of the pre-synaptic pulses. Solid lines are fitted by a double-exponential decay function. EPSC frequency dependence characteristics in (**d**) film-type and (**e**) NW-type chitosan EDL-based synaptic transistors. (**f**) EPSC gains (A_10_/A_1_) according to the pulse frequency.

Figure 3a,b illustrates two consecutive pairs of EPSCs at a fixed V_D_ of 100 mV with 100 ms and 2000 ms inter-pulse intervals in the film- and NW-type devices, respectively. The pulse amplitude and duration were consistently maintained at 1 V and 100 ms, respectively, while the inter-pulse intervals ranged from 3000 ms down to 50 ms. The PPF index, quantified as the ratio of the second EPSC amplitude (A_2_) to the first EPSC amplitude (A_1_) (A_2_/A_1_), was calculated to assess the short-term plasticity in the fabricated film- and NW-type EDL-based synaptic transistors (Figure 3c). As the inter-pulse interval decreased, the PPF index increased, effectively mimicking the short-term plasticity observed in biological synapses. The maximum PPF index reached 132% in the film-type device and 140% in the NW-type device, indicating a faster increase in the PPF index with decreasing pulse intervals in the NW-type device.

The PPF index in biological synapses can be fitted well with a double-exponential decay function, as shown in Equation (1), allowing effective imitation of biological synaptic functionality [33]:

(1) $$PPF\;index=A+C_{1}exp(-\Delta t/\tau_{1})+C_{2}exp(-\Delta t/\tau_{2})$$

where *A* denotes a constant parameter, *C*_1_ and *C*_2_ represent the initial facilitation magnitudes, and *τ*_1_ and *τ*_2_ signify the characteristic relaxation times. The values of relaxation time constants, *τ*_1_ and *τ*_2_, were found to be 175 ms and 896 ms for the film-type device, and 101 ms and 915 ms for the NW-type device, respectively. Furthermore, the proposed device demonstrated the potential to subdivide the synaptic time scale into fast and slow increments, ranging from tens of milliseconds to hundreds of milliseconds.

Moreover, synaptic transistors exhibiting short-term facilitation characteristics can serve as dynamic high-pass temporal filters for signal decoding and enhancement [34]. Figure 3d,e illustrates the frequency dependence of the EPSC in the film- and NW-type synaptic transistors, respectively, stimulated by 10 consecutive pre-synaptic pulses at various frequencies (0.5–9 Hz). The EPSCs generated by sequential spikes exhibited nearly constant values at 1 Hz, progressively increasing with higher frequencies, demonstrating short-term facilitation. The PPF and frequency-dependent facilitation characteristics in EDL-based synaptic transistors arise because the polarized ions from the preceding pulse are not fully depolarized until the arrival of the next pulse, resulting in a stronger polarization [33].

Figure 3f depicts the frequency-dependent EPSC gain, calculated as the ratio of the EPSC triggered by the 10th pre-synaptic spike (A_10_) to the EPSC triggered by the first spike (A_1_). The NW-type synaptic transistor exhibited a remarkable increase in EPSC gain, ranging from 123% to 271%, as the frequency incremented from 0.5 Hz to 9 Hz, outperforming the film-type device at various frequencies. Consequently, the NW channel provides a more efficient synaptic facilitation ability at the same pre-synaptic pulse and frequency.

The proposed synaptic transistor successfully implements the transition from short-term memory (STM) to long-term memory (LTM) through the core element of Atkinson and Shiffrin’s “multi-store model”, which involves repetitive rehearsal (Figure S5). In an electrolyte-based synaptic transistor, the ability to transition to LTM is achieved through an electrochemical doping mechanism, where mobile ions within the electrolyte migrate across the channel interface or are internally inserted [35,36].

**Figure 4:**
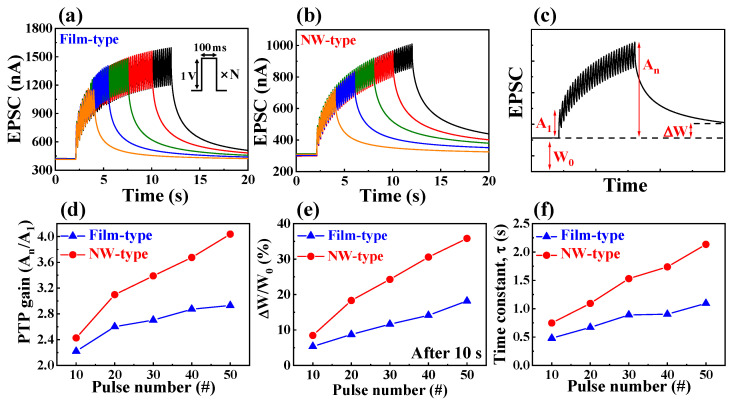
Dynamic retention properties of EPSC in response to multiple pre-synaptic pulses from 10 to 50 in (**a**) film-type and (**b**) NW-type chitosan EDL-based synaptic transistors. (**c**) Essential elements for deriving subsequent parameter values associated with each pre-synaptic pulse. (**d**) Post-tetanic potentiation (PPT) gain (A_n_/A_1_), (**e**) changes in synaptic weight ratio (ΔW/W_0_), and (**f**) retention time constant values according to the number of pulses of film- and NW-type chitosan EDL-based synaptic transistors.

Figure 4a,b illustrates the EPSCs when 10 to 50 consecutive pre-synaptic pulses (1 V, 100 ms) were applied to the film- and NW-type synaptic transistors, respectively. The pulse interval was fixed at 50 ms. Figure 4c shows the elements used for the subsequent parameter calculations, where A_n_ and A_1_ represent the EPSC triggered by the last pulse and the first pulse in consecutive pre-synaptic pulses, respectively. ΔW and W_0_ denote the change in EPSC after 10 s of pulse termination and the initial EPSC, respectively.

As the number of pre-synaptic pulses representing repetitive rehearsal increased, the EPSC value gradually increased, with a more significant increase observed with an increase in the number of rehearsals. When the number of pulses (N) increased from 10 to 50, the post-tetanic potentiation (PTP) gain, calculated by dividing A_n_ by A_1_, increased from 2.42 to 4.04 in the NW-type device and from 2.22 to 2.93 in the film-type device, as shown in Figure 4d. The NW channel exhibited improved PTP gain compared to the film channel.

Furthermore, the device’s memory characteristics and ability to transition between STM and LTM were quantitatively evaluated using the synaptic weight changes before and after pulse application, represented as ΔW/W_0_ [37]. Figure 4e shows the ΔW/W_0_ values according to the number of pre-synaptic pulses. For N = 10, the ΔW/W_0_ values for the film- and NW-type devices were 5.3% and 8.4%, respectively. For N = 50, these values were 18.2% and 35.8%, respectively, indicating that the synaptic weight changes in the NW-type synaptic transistor were approximately twice as large as those in the film-type device at N = 50.

Additionally, the retention time for the decay of the synaptic weight, representing the memory characteristic, was calculated by fitting the decay with the following stretched exponential equation [32]:

(2) G(t)−GinitG0−Ginit=exp[−(tτ)β]

where *G*(*t*), *G_init_*, *G*_0_, *τ*, and *β* are the channel conductance at the time (after the last pulse), initially (before the first pulse), and at the last pulse, retention time, and the stretch index (ranging from 0 to 1), respectively. The fitted curves are shown in Figure S6 (Supporting Information). Figure 4f shows the retention time (*τ*) as a function of the number of pulses. As the number of pulses increased, *τ* increased to 2.13 s at N = 50 in the NW-type devices, which was approximately 2 times longer than the value in the film-type devices (1.09 at N = 50). These results indicate that the transition from STM to LTM was more effectively achieved when the same number of pulses was applied. Therefore, the NW channel not only excels in short-term plasticity (STP), such as PPF, but also in the implementation of long-term plasticity (LTP).

In neural networks, STP and LTP, along with PPF, are essential in the configuration of spiking neural networks (SNNs) [38]. As third-generation neural networks, SNNs are considered the most suitable model for neuromorphic hardware implementations due to their faster processing and highly efficient energy consumption [39,40]. However, compared to the conventional ANN with backpropagation learning, SNNs still lack robust learning rules and their network design principles are immature, requiring further research for commercialization, unlike established frameworks like TensorFlow [41]. Therefore, current artificial synapses require the capability of configuring ANNs with powerful learning abilities, which necessitates the gradual potentiation and depression of synaptic weights in artificial synapses in response to external stimuli [42,43]. Thus, the analog potentiation/depression characteristics of the fabricated film- and NW-type devices were measured. Based on the measured results, the efficiency of constructing an ANN was evaluated through training and recognition simulations using the MNIST hand-written digit database.

**Figure 5:**
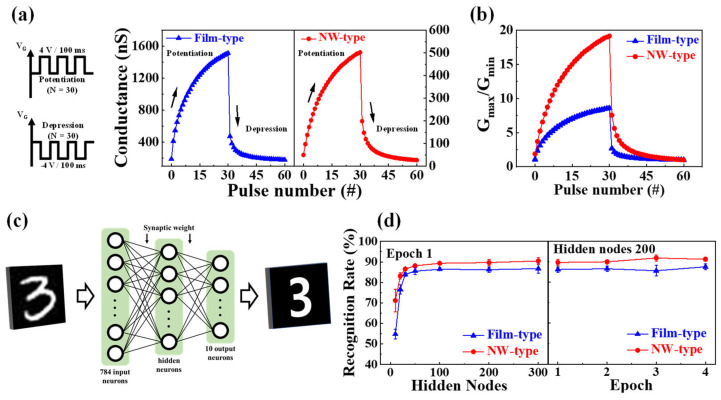
(**a**) Analog change in channel conductance of film- and NW-type chitosan EDL-based synaptic transistors. (**b**) Conductance modulation interpreted by dividing *G_max_* by *G_min_*. (**c**) Schematic diagram of a fully connected artificial neural network (ANN) with three layers (input, hidden, and output) through synaptic weights for Modified National Institute of Standards and Technology (MNIST) simulations. (**d**) Simulated MNIST recognition rates by various numbers of hidden neurons and training epochs.

Figure 5a illustrates the measured analog potentiation/depression characteristics of the film- and NW-type synaptic transistors for ANN construction. Gradual increases in the channel conductance were observed with the application of a positive voltage (4 V, 100 ms), and gradual decreases were observed with the application of negative voltage (−4 V, 100 ms). The channel conductance was extracted through a separate read pulse (0.1 V, 100 ms). These potentiation/depression characteristics, including the maximum/minimum ratio, linearity, and asymmetry ratio (AR), have a significant impact on the performance of the ANN [44].

Figure 5b represents the maximum/minimum conductance (*G_max_*/*G_min_*) of the film- and NW-type devices, which were 8.58 and 19.14, respectively. The NW-type device exhibited approximately 2.23 times higher values, indicating a stronger modulation capability. The AR represents the asymmetry between the potentiation and depression conductance changes, and lower *AR* values indicate more symmetrical conductance changes, leading to increased learning accuracy. The *AR* can be obtained by utilizing the provided equation [45]:

(3) $$AR=\frac{MAX\left|G_{p}\left(n\right)-G_{d}\left(n\right)\right|}{G_{p}\left(30\right)-G_{d}\left(30\right)}\;for\;n=1\;to\;30$$

The *AR*s for the film- and NW-type EDLTs were 0.82 and 0.79, respectively, indicating that the NW type demonstrates a more symmetric conductance change and is closer to the ideal value. To design the ANN model, the normalized conductance and obtained factors were utilized. Equation (4) was employed to model the conductance changes [46]:

(4) G={{(Gmaxα−Gminα)× w+Gminα}1α      if α≠0,Gmin×(Gmax/Gmin)w      if α=0.

where *G_max_* and *G_min_* signify the maximum and minimum values of conductance, respectively, and w is an internal variable that ranges from 0 to 1. In addition, the nonlinear factor α, denoted by α*_p_* for potentiation and α*_d_* for depression, regulates the change in synaptic weight with the aim of an ideal value of 1. For NW-type, α*_p_* and α*_d_* values were calculated to be 3.61 and −2.55, respectively, showing improved linearity of conductance change compared to film-type (α*_p_* = 4.63, α*_d_* = −4.63). The normalized conductance and the obtained factor were utilized in the design of the ANN model.

Figure 5c depicts a schematic diagram of the ANN consisting of three layers: input, hidden, and output. The training process utilized a total of 60,000 28 × 28 MNIST data samples, with a network configuration of 784 input neurons, 10 output neurons, and 10–200 hidden neurons. The characteristics of the weight modulation in the synaptic transistor (channel conductivity) were incorporated into the connectivity between the neurons for the simulation. The simulation was conducted for one epoch, and a sigmoid function was employed as the activation function. The measured channel conductance was normalized as *G*/*G_max_* for the simulation, and a fitting curve using Equation (4) was used.

Figure 5d shows the increase in the recognition rate as the number of hidden nodes increases. The NW-type device exhibited improved potentiation and depression characteristics and factors, resulting in a higher recognition rate compared to the film-type device. The recognition rates of the film- and NW-type EDLTs were 86.2% and 89.6%, respectively, with 300 hidden nodes, and 87.5% and 91.2% after four epochs, respectively. The increase in the number of hidden neurons implies a proportional increase in the number of synaptic transistors in the actual artificial intelligence processor implementation, suggesting that NW-type synaptic devices can achieve higher efficiency with fewer components. Consequently, applying the NW channel structure to the EDL-based synaptic transistor is expected to result in high-performance artificial synaptic devices.

## 4. Conclusions

In this study, we have successfully implemented and characterized chitosan electrolyte-based EDL synaptic transistors with a poly-Si NW channel structure. The addition of the poly-Si NW channel structure has significantly enhanced the performance of the synaptic transistors, making them promising candidates for artificial synaptic devices in neuromorphic computing applications. The formation of the poly-Si NW channel was achieved by transferring the pattern of the electrospun PVP NFs. The NW structure exhibited a high volume-to-area ratio, which enhanced the modulation of the channel conductance through the gate voltage. As a result, the NW-type synaptic transistor demonstrated larger hysteresis window and better performance compared to the film-type one under the same gate voltage. Furthermore, the NW-type synaptic transistor exhibited higher maximum normalized EPSC value, PPF index, and EPSC gain compared to the film-type device under the same pre-synaptic pulse conditions. The effective EPSC modulation capability of the NW channel enabled more efficient transitions from STP to LTP during the rehearsal process, leading to greater synaptic weight changes. These results indicate that the NW-type device is capable of closely mimicking the STP and LTP observed in biological synapses. Analog potentiation/depression characteristics were also measured for potential application in ANNs. The NW-type synaptic transistor, with its superior linearity and maximum-to-minimum conductance ratio (*G_max_*/*G_min_*), achieved higher pattern recognition rates for the MNIST dataset compared to the film-type device in the ANN simulations. This outcome signifies the NW-type device’s suitability for constructing high-performance and low-power ANNs. In conclusion, the incorporation of the poly-Si NW channel structure in chitosan electrolyte-based EDL synaptic transistors has proven highly effective in enhancing their synaptic functionalities. The improved hysteresis window, EPSC modulation capability, and memory transition characteristics make these devices excellent candidates for neuromorphic computing applications, showing potential in the development of efficient and powerful artificial synaptic devices. As neuromorphic hardware advances, these results contribute to the growing field of brain-inspired computing and pave the way for more sophisticated and energy-efficient AI processors. Further research and optimization in this direction hold great promise for future breakthroughs in the fields of AI and neural networks.

## Data Availability

Not applicable.

## Supplementary Materials

The following supporting information can be downloaded at: https://www.mdpi.com/article/10.3390/biomimetics8050432/s1, Figure S1: Schematic illustration of electrospinning equipment; Figure S2: Schematic diagram of the process sequence for poly-Si nanowire channel; Figure S3: (a) Roughness profile and (b) scanning electron microscopy (SEM) image of the poly-Si nanowire (NW) channel; Figure S4: Schematic diagram of proton migration upon applying positive gate voltage in (a) film-type channel and (b) NW-type channel; Figure S5: Schematic illustration of the typical model for transitioning from STM to LTM; Figure S6: Fitting of synaptic weight decay in (a) film-type and (b) NW-type synaptic transistors.
