# Supplementary material for: Enhanced Synaptic Behaviors in Chitosan Electrolyte-Based Electric-Double-Layer Transistors with Poly-Si Nanowire Channel Structures
Source: Biomimetics (Basel). 2023 Sep 18;8(5):432. doi: 10.3390/biomimetics8050432 (PMC10526377; doi:10.3390/biomimetics8050432)
Supplement: Supplementary file 1 [file biomimetics-08-00432-s001.zip › biomimetics-2600448-supplementary.pdf]

Supplementary Information

Enhanced Synaptic Behaviors in Chitosan Electrolyte-Based Electric-Double-Layer Transistors with Poly-Si Nanowire Channel Structures

Dong-Hee Lee[1], Hwi-Su Kim[1], Ki-Woong Park[1], Hamin Park[2] and Won-Ju Cho[1,]*

[1]Department of Electronic Materials Engineering, Kwangwoon University, Seoul 01897, Korea

[2]Department of Electronic Engineering, Kwangwoon University, Seoul 01897, Korea

* Correspondence to: chowj@kw.ac.kr

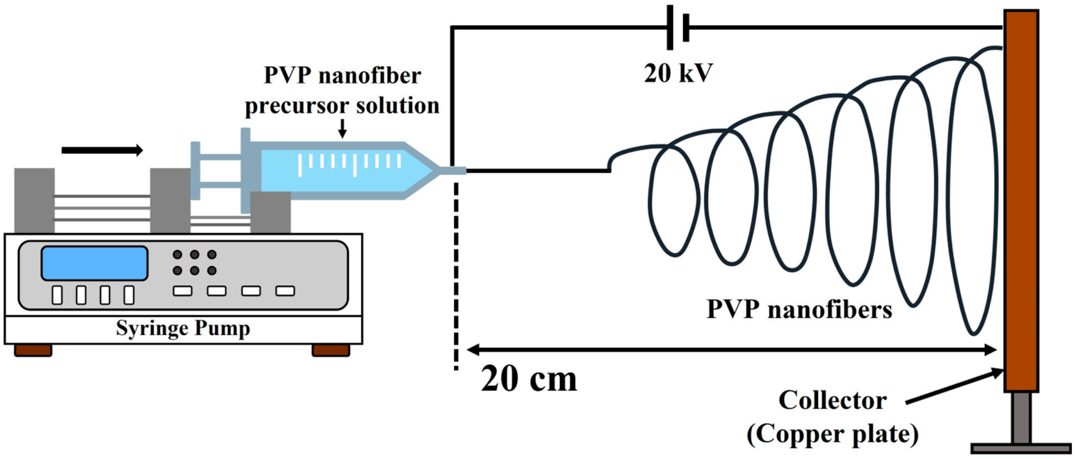

Figure S1. Schematic illustration of electrospinning equipment.

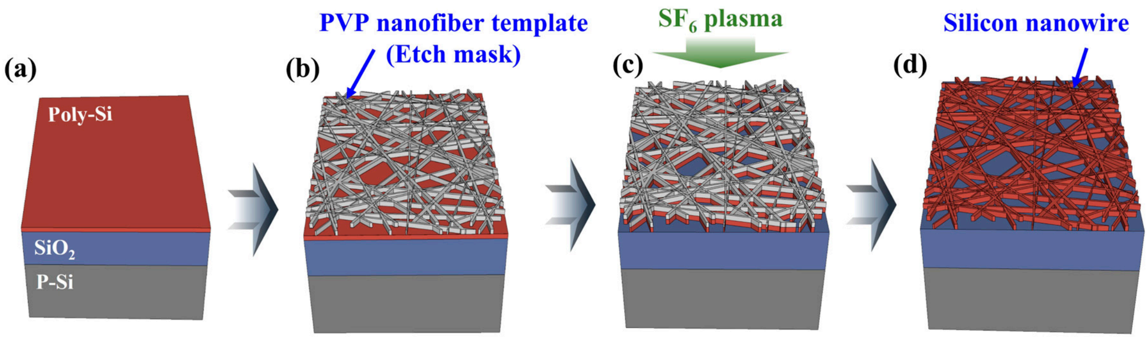

Figure S2. Schematic diagram of the process sequence for poly-Si nanowire channel.

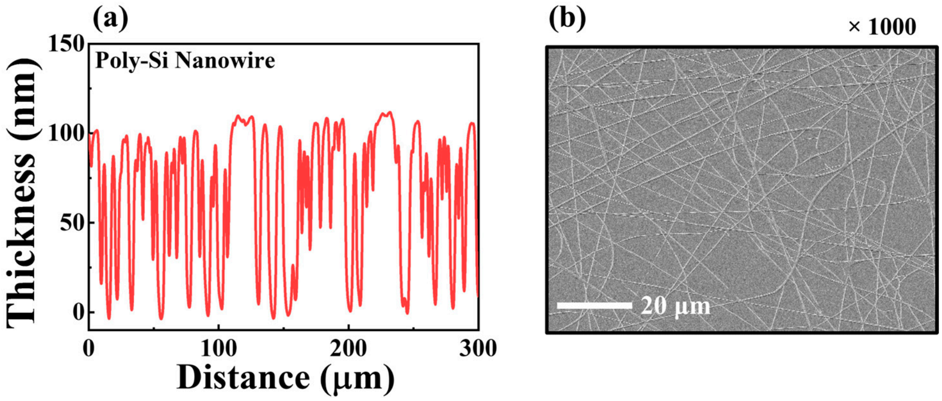

Figure S3. (a) Roughness profile and (b) scanning electron microscopy (SEM) image of the

poly-Si nanowire (NW) channel.

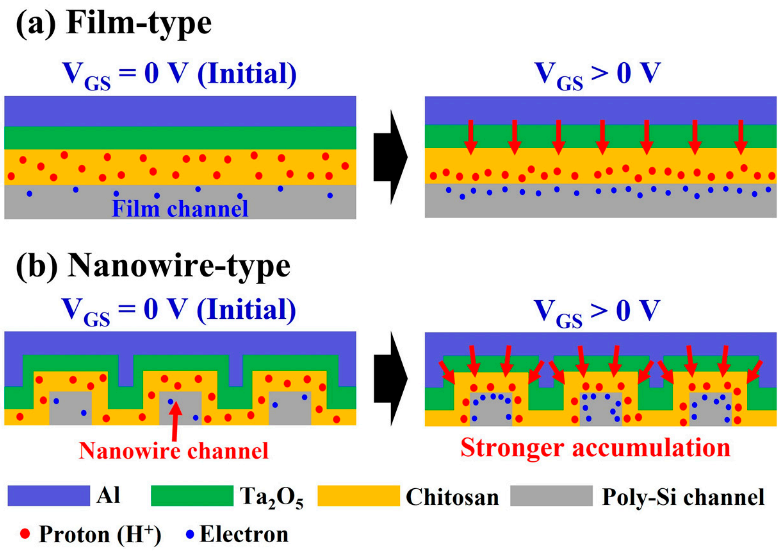

Figure S4. Schematic diagram of proton migration upon applying positive gate voltage in (a) film-type channel and (b) NW-type channel.

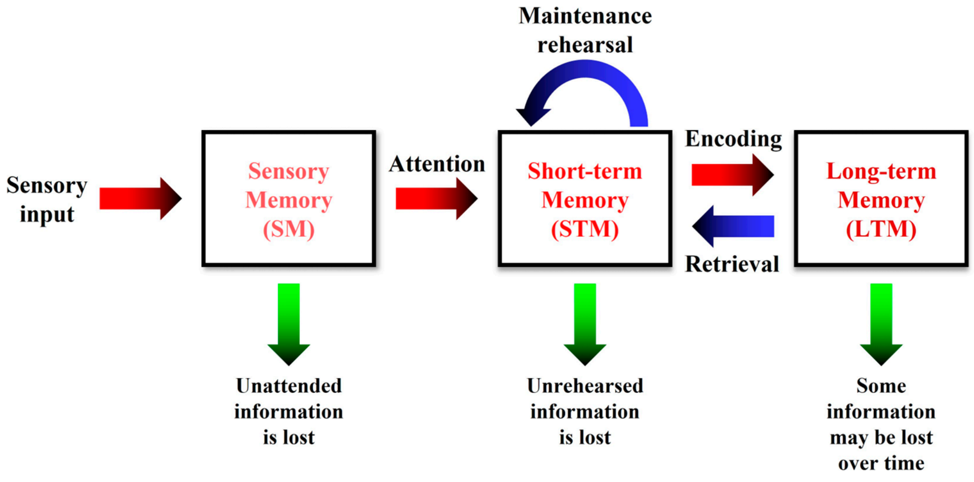

Figure S5. Schematic illustration of the typical model for transitioning from STM to LTM.

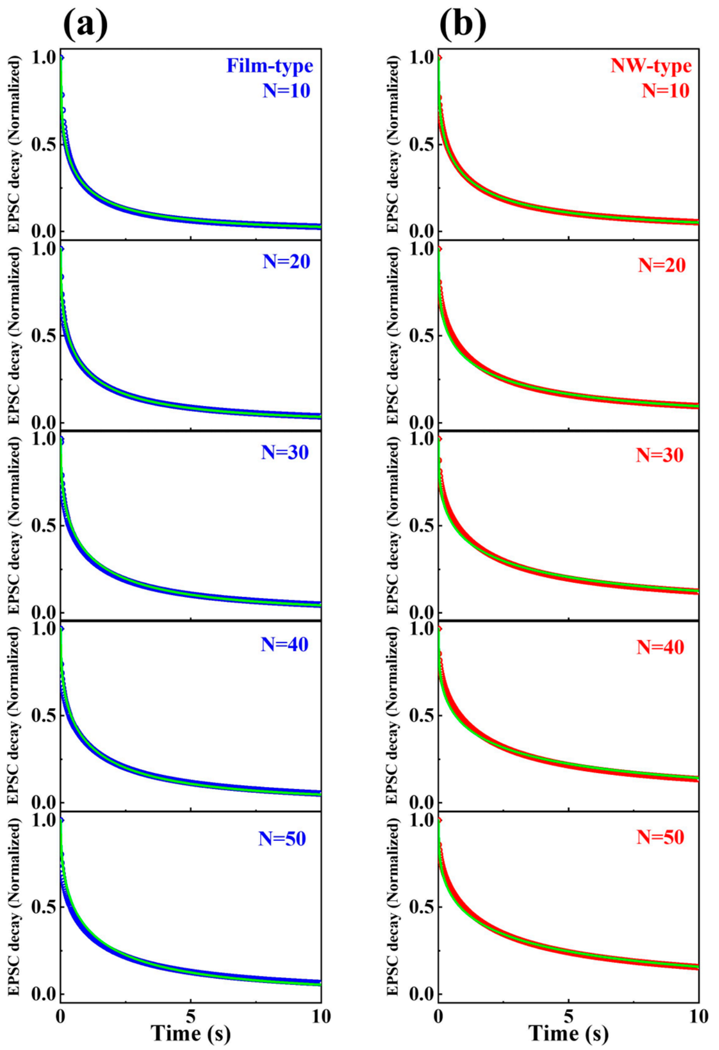

Figure S6. Fitting of synaptic weight decay in (a) film-type and (b) NW-type synaptic transistors.
